# Effectiveness and safety of thymectomy plus prednisone compares with prednisone monotherapy for the treatment of non-thymomatous Myasthenia Gravis: Protocol for a systematic review

**DOI:** 10.1097/MD.0000000000020832

**Published:** 2020-06-19

**Authors:** Huili Yang, Dandan Liu, Xinxin Hong, Haonan Sun, Yu Zheng, Biying Yang, Wanshun Wang

**Affiliations:** a The Second Clinical Medical College of Guangzhou University of Chinese Medicine; b Department of Neurology, Guangdong Provincial Hospital of Chinese Medicine; c Department of Graduate School, Guangzhou University of Chinese Medicine; d General Hospital of Southern Theater Command of PLA, Guangzhou; e The Fourth Clinical Medical College of Guangzhou University of Chinese Medicine, Shenzhen; f Traditional Chinese Medicine Hospital, Xinjiang Medical University, Urumqi, China.

**Keywords:** myasthenia gravis, non-thymomatous, protocol, systematic review, thymectomy plus prednisone

## Abstract

**Background:**

The pathogenesis of myasthenia gravis (MG) has strong connection with thymic abnormalities. Thymic hyperplasia or thymoma can be found with most patients. Thymectomy is currently one of the regular treatment in clinic, which is, however, still controversial for non-thymomatous MG. This research will assess the effectiveness and safety of thymectomy plus prednisone compared to prednisone monotherapy for the treatment of non-thymomatous MG systematically.

**Methods:**

According to eligibility and ineligibility criteria, 8 databases, including PubMed, EMBASE, the Web of Science, the Cochrane Library, China National Knowledge Infrastructure (CNKI), Wan-fang Database, Chinese Biomedical Literature Database (CBM), China Science and Technology Journal Database (CSTJ), will be searched to gather the up-to-standard articles from September 2000 to September 2025. Inclusion criteria are as follows: randomized controlled trials of thymectomy plus prednisone for the treatment of non-thymomatous MG. The quantitative myasthenia gravis score (QMG) and the dose of prednisone required will be accepted as the main outcomes. Data synthesis, subgroup analysis, sensitivity analysis, and meta-regression analysis will be conducted using RevMan 5.3 software. We will use Egger or Begg test to evaluate symmetry on a funnel plot which is made to assess reporting bias, and use trial sequential analysis (TSA) to exclude the probability of false positives.

**Results:**

This systematic review will measure the QMG and the dose of prednisone required, the myasthenia gravis activities of daily living scale scores (MG-ADL), treatment-associated complications, incidence of myasthenic crisis and other aspects to comprehensively assess the clinical benefits of thymectomy plus prednisone for MG patients without thymoma.

**Conclusion:**

The conclusion of this study will achieve convincing evidence to evaluate the effectiveness and safety of thymectomy plus prednisone for the treatment of non-thymomatous MG.

**PROSPERO registration number:**

CRD 42020167735.

## 1 Introduction

Myasthenia gravis (MG) is a neuromuscular and autoimmune disease caused by acetylcholine receptor antibody (AChRAb), of which the pathogenesis is humoral immunity, cellular immunity, and complement involved together.^[1,2]^ The global incidence and the annual incidence of MG, is 150 to 250 and 8 to 10 per 1 million, respectively.^[3]^ About 70% MG patients have thymus hyperplasia, and ∼10% MG patients can be found thymoma.^[4]^ Glucocorticoid, cholinesterase inhibitor, immunosuppressant, intravenous immunoglobulin, and plasma exchange are common medical treatments for MG, while thymectomy is a surgical therapy of MG.

As one of the important clinical therapeutic drugs, prednisone is usually applied for the drug therapy of MG by inhibiting immune response.^[5]^ Although the drug treatment can relieve and stabilize the condition of the most MG patients with thymus hyperplasia but without thymoma, there are still some patients, who have a relapse, myasthenic crisis, even severity adverse drug reaction including peptic ulcer, osteoporosis, pathological fracture, osteonecrosis of the femoral head, myelosuppression, etc.^[1]^ Thymic pathological changes can activate the autoimmune response towards acetylcholine receptor, whereas thymusectomy will reduce the sources of abnormal immunity. Furthermore, thymusectomy can relieve the condition and reduce the dosage of immunosuppressant, especially for patients whose disease classification are refractory generalized myasthenia gravis (GMG) or who have had myasthenic crisis.^[6]^ Thymectomy has become an important therapy for the MG patients with thymoma, of which the effectiveness has been widely recognized.^[7,8]^ Recent research suggested that thymusectomy could raise the remission rate of the MG patients, no matter with or without thymomatous.^[9]^ In the multi-center randomized controlled clinical trials, researchers believe that the clinical benefits of thymectomy plus prednisone are better than prednisone monotherapy for non-thymomatous MG.^[10,11]^ However, because of unavoidable post-operative complications and inconsistency of clinical outcomes in different studies,^[12]^ thymectomy is still controversial for non-thymomatous MG. In addition, there is few clinical evidence can prove that thymectomy has prominent clinical efficacy and safety for the MG patients without thymoma.

To the best of our knowledge, current systematic reviews barely involve randomized controlled trials (RCTs) in Chinese database. Besides, there is a lack of systematic reviews to definitely confirm the clinical effect of thymectomy plus prednisone for non-thymomatous MG. Accordingly, this research is aimed to systematically state the effectiveness and safety of thymectomy plus prednisone for non-thymomatous MG by summarizing results of the published clinical trials, and provide theoretical basis or guidance for the future research and clinical treatment.

## 2 Methods

### 2.1 Registration

The protocol of this study has been registered in the international prospective register of systematic reviews (PROSPERO). The registration number of PROSPERO is CRD 42020167735.

### 2.2 Ethics and dissemination

The data we needed comes from published researches, which has no direct connection with patients’ individual data. Thus, an ethical approval is not required. The achievements of this systematic assessment will give implication of the efficacy and safety of thymectomy plus prednisone for non-thymomatous MG and be published in a peer-reviewed journal, which can help clinicians make better clinical decisions.

### 2.3 Eligibility criteria

#### 2.3.1 Participants

Patients will be included when meet the following items: onset within 5 years; age 18 to 65 years; acetylcholine-receptor-antibody level over 1.00 nmol/L or 0.50 to 0.99 nmol/L if edrophonium test is positive, repetitive nerve stimulation or single-fiber electromyography is abnormal; fit the MG recommendations for clinical research standards of II to IV.^[13]^ Any patients who suffer from thymoma on CT or MRI of the chest, thymectomy history, pregnancy, breastfeeding, contraindications to glucocorticoids or serious illness will be excluded.

#### 2.3.2 Interventions

The therapy of the experimental group was thymectomy (through the median sternotomy for resecting all the mediastinal tissue entirely) plus prednisone while the control group was given prednisone monotherapy in eligible studies.

#### 2.3.3 Outcome

The main outcome measures are the time-weighted average (TWA) quantitative myasthenia gravis score (QMG)^[14]^ and the TWA needed dose of prednisone by assessing in each stage. The myasthenia gravis activities of daily living scale scores (MG-ADL),^[15,16]^ treatment-associated complications, incidence of myasthenic crisis and the mortality over the follow up period are defined as the secondary outcomes.

#### 2.3.4 Study design

All RCTs of thymectomy plus prednisone for non-thymomatous MG that have control groups will be included, without restriction of language, published status, and sample size.

### 2.4 Ineligibility criteria

We will exclude non-RCTs included observational researches, case reports, repeated reports, conference articles, and reviews.

### 2.5 Search strategy

All the studies will be searched on the basis of eligibility criteria from eight databases consisted of PubMed, EMBASE, the Web of Science, the Cochrane Library, China National Knowledge Infrastructure (CNKI), Wan-fang Database, Chinese Biomedical Literature Database (CBM), China Science and Technology Journal Database (CSTJ) from September 2000 to September 2025. P+I+C+O+S, P+I+C+O and P+I+O will be used for searched topics to make sure the accuracy rate of literature retrieval. All the search terms from article will be listed in the controlled vocabulary. Also, the fields of title, abstract and keyword will be selected according to the characteristics of different databases. The search items are divided into three blocks and displayed in Table 1.

**Table 1:**
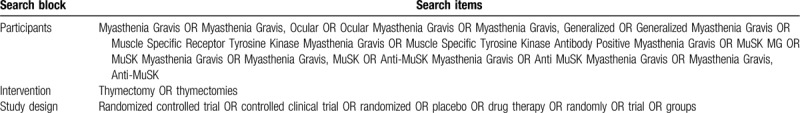
Search items.

### 2.6 Study selection and data extraction

#### 2.6.1 Study selection

**Figure 1:**
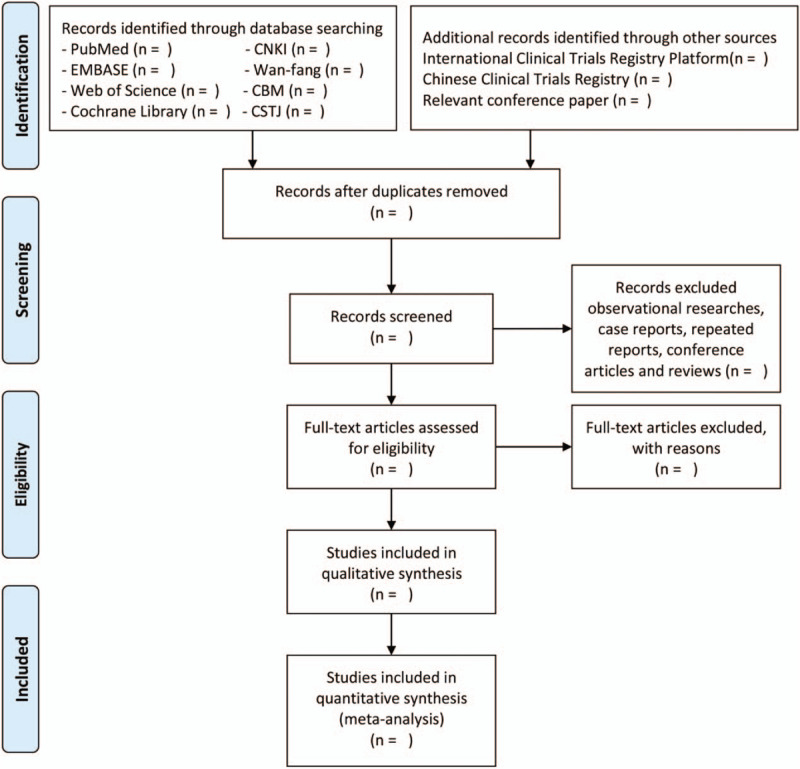
PRISMA flow diagram of study selection process in the systematic review.

Two researchers will be in charge of preliminary screening by titles and abstracts independently and remove duplicate data as well as obviously ineligible literatures. Then, the third researcher will estimate the full text of divergent literatures and organize group discussion to gain consensus. In the second filter, ineligible reasons of the excluded literatures will be recorded. The selection process of eligible papers is presented by a preferred reporting items for systematic review and meta-analysis (PRISMA) flow diagram (Fig. 1).

#### 2.6.2 Data extraction

The data including publication time, the first author, sample size, gender, mean age, diagnostic information, duration, specifics of intervention, and control group, specifics of outcomes and various adverse events from eligible literatures will be extracted by two reviewers independently while the third reviewer, as an arbitrator, will evaluate all the data and divergence.

#### 2.6.3 Processing missing data

If the abovementioned data is insufficient, the primary authors of studies will be contacted through e-mail or telephone in order to gain missing data. We will add underlying impact of unavailable data in the discussion part when missing data cannot be acquired.

### 2.7 Risk of bias assessment

Two reviewers will evaluate the risk of bias of all the included articles through the Cochrane Collaboration's tool,^[17]^ respectively. Stochastic sequence generation, allocation concealment, blinding of participants, blinding of personnel, blinding of outcome, fragmentary outcome data, selective reporting and other possible sources of bias are details of the assessment. The above items will be graded as “high risk,” “low risk,” or “unclear risk.” If divergence appears, the third reviewer will resolve by organizing discussion.

### 2.8 Data synthesis

We will calculate the data synthesis by means of RevMan 5.3 software. A risk ratio with 95% confidence intervals (CI) will be used to express the clinical efficiency, and odds ratio with 95% CI will signify incidence of adverse events, while weighted mean difference with 95% CI will show clinical scores before and after the treatment. The *I*-squared statistic will be computed to evaluate the heterogeneity between all the researches.^[18]^*I*^2^ <50% is regarded as no significant heterogeneity, then the fixed-effect model will be used. However, *I*^2^ ≥50% means significant heterogeneity, and we will combine the results of studies by the random-effect model.

### 2.9 Subgroup analysis, sensitivity analysis, and meta-regression analysis

If there exists substantial heterogeneity in included studies, we will investigate the possible sources of heterogeneity through subgroup analysis, sensitivity analysis or meta-regression analysis. Subgroup analysis includes following main items: patients’ age, onset time, duration of disease, severity of phenotype, dose of prednisone formulas and other related parameters. We will perform sensitivity analysis including methodological quality, researches quality, sample characteristic, heterogeneity, impact of missing data, etc. And then the reliability of sensitivity analysis will be assessed. In case of insufficiency of data exaction, a qualitative synthesis will be built.

### 2.10 Assessment of reporting biases

In case the quantity of pooled researches is enough (n ≥ 10), we will use Egger or Begg test to evaluate symmetry on a funnel plot which is made to assess reporting bias.

### 2.11 Test sequential experiment

Sample size analysis has been conducted through trial sequential analysis (TSA)^[19,20]^ to exclude the probability of false positives, and also demonstrated the dependability of our analysis results.

### 2.12 Quality of evidence

The quality of evidence will also be evaluated through The Grading of Recommendations Assessment, Development, and Evaluation (GRADE)^[21]^ approach. We will investigate limitations of study design, inaccuracies, inconsistencies, indirectness as well as reporting biases, and grade the level of evidence into very low, low, moderate, or high four levels.

## 3 Discussion

MG is a lifelong autoimmune disease, for which, prednisone and azathioprine are the first line treatment drugs, among immunosuppressant,^[22]^ which can induce endocrine disorders, centripetal obesity, rarefaction of bone, osteonecrosis of femoral head and other adverse reactions in the case of long-term treatment.^[1]^ About 10% MG patients have thymoma who should be treated with thymectomy unless inoperable. More than 70% of MG patients have thymus hyperplasia, but only those having severe symptoms are thought to gain more clinical benefit from thymectomy. To patients without thymoma, thymectomy may be an approach that can reduce the dosage or time of using immunosuppressant potentially, especially for those who are intolerant of adverse reactions or cannot respond well to immunotherapy.^[10,23]^ Many clinical studies have demonstrated that patients without thymoma can also gain good effect within 2 to 24 months after thymectomy, yet the risk of postoperative myasthenic crisis, pain, infection, and recrudescence makes this therapy controversial.^[24–27]^ In fact, if there is adequate evidence to prove the advantages of thymectomy, that would be a good news to patients without thymoma. So far, the clinical evidence about thymectomy plus prednisone compared to prednisone monotherapy for non-thymomatous MG has not been evaluated systematically. Thus, we decide to evaluate the effectiveness and safety of thymectomy plus prednisone through this study and hope to provide more clinical evidence about thymectomy for non-thymomatous MG. Nevertheless, it is almost impossible to avoid limitations, for instance, publishing bias and heterogeneity, in this systematic review, so it is essential for us to analyze more researches and results unpublished.

## Author contributions

Huili Yang and Wanshun Wang designed the study. Huili Yang, Dandan Liu and Xinxin Hong drafted the protocol. All authors revised this manuscript and confirmed the final version.

**Conceptualization:** Huili Yang, Wanshun Wang.

**Data curation:** Huili Yang, Dandan Liu, Xinxin Hong, Haonan Sun, Wanshun Wang.

**Formal analysis:** Huili Yang, Dandan Liu.

**Investigation:** Xinxin Hong, Haonan Sun, Yu Zheng.

**Methodology:** Huili Yang, Dandan Liu, Xinxin Hong, Wanshun Wang.

**Project administration:** Huili Yang, Wanshun Wang.

**Resources:** Wanshun Wang.

**Software:** Huili Yang.

**Supervision:** Wanshun Wang.

**Validation:** Wanshun Wang.

**Visualization:** Huili Yang, Dandan Liu, Biying Yang.

**Writing – original draft:** Huili Yang, Dandan Liu.

**Writing – review & editing:** Huili Yang, Wanshun Wang.

## References

[1] Gilhus NE Verschuuren JJ Myasthenia gravis: subgroup classification and therapeutic strategies. Lancet Neurol 2015;14:1023–36. 26376969 10.1016/S1474-4422(15)00145-3

[2] Gilhus NE Myasthenia gravis. N Engl J Med 2016;375:2570–81. 28029925 10.1056/NEJMra1602678

[3] Carr AS Cardwell CR McCarron PO A systematic review of population based epidemiological studies in Myasthenia Gravis. BMC Neurol 2010;10:46. 20565885 10.1186/1471-2377-10-46 PMC2905354

[4] Barnett C Katzberg HD Keshavjee S Thymectomy for non-thymomatous myasthenia gravis: a propensity score matched study. Orphanet J Rare Dis 2014;9:214. 25539860 10.1186/s13023-014-0214-5 PMC4296689

[5] Farmakidis C Pasnoor M Dimachkie MM Treatment of myasthenia gravis. Neurol Clin 2018;36:311–37. 29655452 10.1016/j.ncl.2018.01.011 PMC6690491

[6] Melzer N Ruck T Fuhr P Clinical features, pathogenesis, and treatment of myasthenia gravis: a supplement to the Guidelines of the German Neurological Society. J Neurol 2016;263:1473–94. 26886206 10.1007/s00415-016-8045-z PMC4971048

[7] Ruffini E Guerrera F Filosso PL Extended transcervical thymectomy with partial upper sternotomy: results in non-thymomatous patients with myasthenia gravis. Eur J Cardio-Thorac 2015;48:448–54. 10.1093/ejcts/ezu442 25428934

[8] Souto EB Lima B Campos JR Myasthenia gravis: State of the art and new therapeutic strategies. J Neuroimmunol 2019;577080. 31670062 10.1016/j.jneuroim.2019.577080

[9] De Roxas RC Bagnas MAC Baldonado JJAR Clinical profile and outcome of postthymectomy versus non-thymectomy myasthenia gravis patients in the Philippine general hospital: a 6-year retrospective study. Front Neurol 2016;7:96. 27445963 10.3389/fneur.2016.00096 PMC4914503

[10] Wolfe GI Kaminski HJ Aban IB Randomized trial of thymectomy in myasthenia gravis. N Engl J Med 2016;375:511–22. 27509100 10.1056/NEJMoa1602489 PMC5189669

[11] Wolfe GI Kaminski HJ Aban IB Long-term effect of thymectomy plus prednisone versus prednisone alone in patients with non-thymomatous myasthenia gravis: 2-year extension of the MGTX randomised trial. Lancet Neurol 2019;18:259–68. 30692052 10.1016/S1474-4422(18)30392-2 PMC6774753

[12] Cea G Benatar M Verdugo RJ Thymectomy for non-thymomatous myasthenia gravis. Cochrane Database Syst Rev 2013;CD008111. 24122674 10.1002/14651858.CD008111.pub2 PMC12083877

[13] Jaretzki A Barohn RJ Ernstoff RM Myasthenia gravis: recommendations for clinical research standards. Neurology 2000;55:16–23. 10891897 10.1212/wnl.55.1.16

[14] Bedlack R Simel D Bosworth H Quantitative myasthenia gravis score: assessment of responsiveness and longitudinal validity. Neurology 2005;64:1968–70. 15955957 10.1212/01.WNL.0000163988.28892.79

[15] Wolfe GI Herbelin L Nations S Myasthenia gravis activities of daily living profile. Neurology 1999;52:1487–1487. 10227640 10.1212/wnl.52.7.1487

[16] Muppidi S Wolfe GI Conaway M MG-ADL: still a relevant outcome measure. Muscle Nerve 2011;44:727–31. 22006686 10.1002/mus.22140

[17] Higgins JP Altman DG Gøtzsche PC The Cochrane Collaboration's tool for assessing risk of bias in randomised trials. BMJ 2011;343:d5928. 22008217 10.1136/bmj.d5928 PMC3196245

[18] Higgins JP Thompson SG Deeks JJ Measuring inconsistency in meta-analyses. BMJ 2003;327:557–60. 12958120 10.1136/bmj.327.7414.557 PMC192859

[19] Wetterslev J Thorlund K Brok J Trial sequential analysis may establish when firm evidence is reached in cumulative meta-analysis. J Chronic Dis 2008;61:64–75. 10.1016/j.jclinepi.2007.03.013 18083463

[20] Thorlund K Engstrøm J Wetterslev J User manual for trial sequential analysis (TSA). Copenhagen Trial Unit Centre Clin Intervent Res 2011;1:1–15.

[21] Puhan MA Schünemann HJ Murad MH A GRADE Working Group approach for rating the quality of treatment effect estimates from network meta-analysis. BMJ 2014;349:g5630. 25252733 10.1136/bmj.g5630

[22] Sanders DB Wolfe GI Benatar M International consensus guidance for management of myasthenia gravis: executive summary. Neurology 2016;87:419–25. 27358333 10.1212/WNL.0000000000002790 PMC4977114

[23] Kim SW Choi Y-C Kim SM Effect of thymectomy in elderly patients with non-thymomatous generalized myasthenia gravis. J Neurol 2019;266:960–8. 30726532 10.1007/s00415-019-09222-2

[24] Aydin Y Ulas AB Mutlu V Thymectomy in myasthenia gravis. Eurasian J Med 2017;49:48. 28416933 10.5152/eurasianjmed.2017.17009 PMC5389494

[25] Kadota Y Horio H Mori T Perioperative management in myasthenia gravis: republication of a systematic review and a proposal by the guideline committee of the Japanese Association for Chest Surgery 2014. Gen Thorac Cardiovas 2015;63:201–15. 10.1007/s11748-015-0518-y 25608954

[26] Yu S Li F Chen B Eight-year follow-up of patients with myasthenia gravis after thymectomy. Acta Neurol Scand 2015;131:94–101. 25170783 10.1111/ane.12289

[27] Yang J Liu C Li T Prognosis of thymectomy in myasthenia gravis patients with thymus hyperplasia. Int J Neurosci 2017;127:785–9. 27819773 10.1080/00207454.2016.1257993

